# *In vivo* development of cefiderocol resistance in carbapenem-resistant *Acinetobacter baumannii* associated with the downregulation of a TonB-dependent siderophore receptor, PiuA

**DOI:** 10.1093/jac/dkae018

**Published:** 2024-01-31

**Authors:** Jacqueline Findlay, Gabriele Bianco, Matteo Boattini, Patrice Nordmann

**Affiliations:** Medical and Molecular Microbiology, Faculty of Science and Medicine, University of Fribourg, Fribourg, Switzerland; Microbiology and Virology Unit, University Hospital Città della Salute e della Scienza di Torino, Turin, Italy; Department of Public Health and Paediatrics, University of Torino, Turin, Italy; Microbiology and Virology Unit, University Hospital Città della Salute e della Scienza di Torino, Turin, Italy; Department of Public Health and Paediatrics, University of Torino, Turin, Italy; Medical and Molecular Microbiology, Faculty of Science and Medicine, University of Fribourg, Fribourg, Switzerland; Swiss National Reference Center for Emerging Antibiotic Resistance (NARA), University of Fribourg, Fribourg, Switzerland

Carbapenem-resistant *Acinetobacter baumannii* (CRAB) are a leading cause of nosocomial infections and subsequently present an urgent global public health threat.^1^ OXA-23 is the most frequently reported carbapenemase in CRAB and such isolates are most often multidrug-resistant, leaving few therapeutic options.^1^ Cefiderocol (FDC), a recently approved siderophore cephalosporin, represents a promising treatment option for CRAB infections, particularly since reported resistance rates in non-metallo-beta-lactamase-producing CRAB remain low.^2^ FDC resistance in *A. baumannii* has previously been reported to be associated with mutations and/or deletions in the TonB-dependent receptors PiuA and PirA,^3^ the carriage of certain beta-lactamases (e.g. MBLs and PER-type),^4^ and mutations within the penicillin binding protein PBP-3, the main target of FDC.^3^ In this study, we describe the *in vivo* development of FDC resistance following treatment, mediated by a previously undescribed mutation in the promoter region of TonB-dependent receptor, PiuA.

An 80-year-old woman was admitted to the ICU of the University Hospital ‘Città della salute e della scienza di Torino’ (Turin, Italy) due to a severe burn injury (20% TBSA). Empirical antibiotic therapy with piperacillin/tazobactam plus amikacin was started. On day 52, she presented with fever and elevation of inflammatory markers. Blood cultures were positive for an FDC-susceptible CRAB isolate (AB1). FDC plus fosfomycin were started and the patient clinically improved. After 10 days, she presented with fever and an FDC-resistant CRAB isolate was isolated from a blood culture (AB2). FDC treatment was stopped, fosfomycin plus ampicillin/sulbactam were started, and the patient clinically improved. Susceptibility testing, performed by broth microdilution and interpreted according to EUCAST guidelines,^5^ showed that isolates AB1 and AB2 exhibited FDC MICs of 1 and 32 mg/L respectively, and were additionally resistant to both imipenem and meropenem (Table 1). Isolates were subject to Illumina WGS and subsequent analyses were performed using the Center for Genomic Epidemiology Platform (https://www.genomicepidemiology.org/) as previously described.^6^ Both isolates belonged to ST2 (according to the Pasteur MLST scheme)^7^ and harboured an identical resistance gene content, including a carbapenemase encoding gene *bla*_OXA-23_ and the intrinsic beta-lactamase genes *bla*_OXA-66_ and *bla*_ADC-293_. The isolates additionally harboured a gene encoding the aminoglycoside-modifying enzyme ArmA. ST2 *A. baumannii* is the most dominant type globally (a member of Global Clone 2) and is considered a high-risk clone, often associated with the production of carbapenemases, particularly OXA-23.^1^ SNP analyses using Snippy (https://github.com/tseemann/snippy) identified that the only difference between both isolates was a 3 nt deletion, located 35 bp upstream of a TonB-dependent siderophore receptor gene, *piuA*. This deletion was located between the −35 and −10 elements of a predicted σ70 promoter, resulting in a ‘weaker’ promoter sequence, predicted to result in decreased RNA polymerase binding. To investigate the impact of this deletion on gene expression quantitative RT–QPCR was performed on both isolates, AB1 and AB2. Briefly, isolates were grown in iron-depleted Mueller–Hinton media to mid-exponential growth phase (OD_600_ ∼0.5), before RNA extraction using the Monarch^®^ Total RNA Miniprep Kit (New England Biolabs), DNA digestion using TURBO^™^ DNase (ThermoFisher) and cDNA synthesis using LunaScript (New England Biolabs). Quantitative PCR was performed on a Qiagen RotorGene using the Promega SYBR green PCR mix and the following primers: for *piuA*, AB_piuA_qF (5′-CAGTTGGTGGCAGCATCAAT-3′) and AB_piuA_qR (5′-TGCTGCAATGCCATTTCCAA-3′), and for *rpoB*, AB_rpoB_qF (5′-ACGCCTAAAGGTGAAACTCAGTTAA-3′) and AB_rpoB_qR (5′-GTACCAGATGGAACACGTAAAGATG-3′). All assays were performed on three biological replicates, and the delta delta Ct method was used to calculate relative expression levels. Analyses identified that the *piuA* was significantly downregulated (∼3-fold reduction; Table 1) in isolate AB2, and subsequently this was determined to be the mechanism responsible for elevated the FDC MICs. To investigate the fitness cost of this insertion, growth curves were performed on three biological replicates for each isolate, in iron-depleted Mueller–Hinton media over an 8-hour period. This showed the promoter mutation in strain AB2 resulted in a significant fitness cost, ranging from a 15% to 30% reduction in growth, over the early to late exponential growth phase (OD_600_ 0.3–0.8), relative to AB1. This can be explained by the fact that iron is essential for cell function, and the limited uptake through a functional PiuA could be expected to compromise cell fitness.^8^

**Table 1.** Genotypic and phenotypic characteristics of isolates AB1 and AB2

				MICs (mg/L)				
Isolate	ST	Beta-lactamase genes	Other resistance genes	FDC	IPM	MEM	SNPs	Relative *piuA* expression
AB1	2	*bla*_OXA-23_, *bla*_OXA-66_, *bla*_ADC-293_	*armA*, *aph(3’)-Ib*, *aph(6)-Id*, *msr(E)*, *mph(E)*, *sul2*, *tetB*	1	128	64	NA	1
AB2	2	*bla*_OXA-23_, *bla*_OXA-66_, *bla*_ADC-293_	*armA*, *aph(3’)-Ib*, *aph(6)-Id*, *msr(E)*, *mph(E)*, *sul2*, *tetB*	32	128	64	3 nt deletion upstream of *piuA*	0.35

FDC, cefiderocol; IPM, imipenem; MEM, meropenem.

In conclusion, this study illustrated the *in vivo* development of FDC resistance in an OXA-23-producing ST2 *A. baumannii* isolate, as a direct consequence of FDC therapy. A single mutation event was sufficient to result in a 32-fold increase in FDC MIC although this resistance was shown to come at a fitness cost to the bacterium. This demonstrates the ability of *A. baumannii* to mutate and adapt to cope with challenge by FDC, and underlines that despite being a promising therapeutic option for the treatment of CRAB, FDC should be prescribed sparingly to preserve its use. Studies on the evaluation of the most effective combinations for treating infections due to multidrug-resistant *A. baumannii* infections are ongoing. They include drugs such as FDC, ampicillin/sulbactam sulbactam/durlobactam colistin and rifampicin. The use of FDC in combination with another antibiotic is debatable for preventing emerging resistance to FDC, and certainly requires further investigation.
